# Association between anticholinergic activity and xerostomia and/ or xerophthalmia in the elderly: systematic review

**DOI:** 10.1186/s40360-022-00637-8

**Published:** 2022-12-21

**Authors:** E. Prado-Mel, P. Ciudad-Gutiérrez, H. Rodríguez-Ramallo, S. Sánchez-Fidalgo, B. Santos-Ramos, A. M. Villalba-Moreno

**Affiliations:** 1 Hospital Universitario Vírgen del Rocío(Pharmacy department), Andalucía, Seville, Spain; 2 University of Seville(Department of Preventive Medicine and Public Health), Andalucía, Seville, Spain

**Keywords:** Anticholinergic burden, Anticholinergic scale, Anticholinergic drugs, Xerophthalmia, Xerostomia

## Abstract

**Purpose:**

The aim of this work was to investigate the association between anticholinergic burden or anticholinergic drug use and xerostomia and/or xerophtalmia in elderly through a systematic review of the published literature.

**Methods:**

A search was carried out in 3 databases (CINAHL, Embase and Pubmed). Studies conducted in people ≥65 years of age, who took anticholinergic medications, and measured the association between the anticholinergic burden or the use of these medications with the prevalence of xerostomia and / or xerophthalmia, published up to August 2022, were selected. Studies published in languages other than Spanish and/or English were excluded.

**Results:**

One thousand two hundred eleven articles were identified, 10 were selected for this review: six cross-sectional studies, two cohorts, one case-control and one randomized controlled clinical trial. A total of 3535 patients included in the different studies were studied. The most used scales were the Anticholinergic Drug Scale (ADS) and the Anticholinergic Risk Scale (ARS). Four articles studied the relationship between the use of anticholinergic medication and the prevalence of xerostomia and / or xerophthalmia, finding a positive relationship with xerostomia in all of them. Another 6 measured the relationship between anticholinergic burden and xerostomia and / or xerophthalmia. Four found a positive relationship between anticholinergic burden and xerostomia and/or xerophthalmia.

**Conclusions:**

Our findings suggest a clear relationship between the use of anticholinergic drugs or anticholinergic burden and the presence of xerostomia. This relationship was less conclusive in the case of xerophthalmia.

**Supplementary Information:**

The online version contains supplementary material available at 10.1186/s40360-022-00637-8.

## Introduction

The use of medications in patients older than 65 is high, with 20% of this population estimated to take 5 on average [1]. Currently, almost 50% of elderly patients take at least one medication with anticholinergic activity [2]. Various authors have highlighted the particular vulnerability of the elderly to the effects of such drugs. It is known that drugs metabolism and excretion are reduced in this population, leading to a higher risk of toxicity [3, 4]. At the central nervous system level, there is evidence that suggests anticholinergic-related cognitive impairment, delirium and dementia [5]. As for the peripheral nervous system, anticholinergics are linked to effects that can be underestimated on many occasions by health professionals, including, but not limited to secretions, constipation, increased heart rate, xerostomia and xerophthalmia [4].

The anticholinergic toxicity associated with the cumulative effect of one or more medications with anticholinergic activity is referred to as “anticholinergic burden.” [6] Anticholinergic burden in the elderly is associated with flare-ups or the worsening of certain chronic health conditions, which lead to increased hospitalizations, care burden and even mortality [7].

Xerostomia is defined as a subjective feeling of dry mouth, and even though most authors relate xerostomia to a lack of salivary secretion (hyposalivation), this is not true for every case [8, 9]. Both symptoms are associated with people aged over 60 years, polypharmacy and coexistence of conditions [10] such as diabetes and rheumatic diseases like rheumatoid arthritis or Sjögren syndrome, but they can also be a lesser studied adverse effect of anticholinergics. Consequently, xerostomia put patients at a higher risk of oropharyngeal infection, tooth loss, dysphagia, dysgeusia, chewing problems and even nocturnal discomfort due to dry mouth [8, 11, 12].

Xerophthalmia is the eye’s inability to produce enough tears to stay adequately lubricated, leading to stinging, discomfort, mydriasis and visual accommodation problems [13]. Xerophthalmia is also linked to diseases such as diabetes, rheumatoid arthritis, Sjögren syndrome, as well as eye surgery or nutritional deficiencies, including vitamin A deficiency [14]. Both xerostomia and xerophthalmia profoundly affect patients’ quality of life and have the potential to trigger more severe clinical situations [15]. In older patients, tooth loss coupled with anticholinergic drug-induced xerostomia can lead to malnutrition and/or sarcopenia that could contribute to the worsening of underlying chronic conditions [16].

Previous reviews searching for a causal relationship between anticholinergic burden and adverse outcomes have focused primarily on central anticholinergic adverse effects [17–19]. Thus, the association with peripheral adverse events such as xerostomia and xerophthalmia has not been clearly defined or documented.

Therefore, it is necessary to systematically understand the evidence on the connection between the use of anticholinergics and peripheral adverse effects, including xerostomia and xerophthalmia. To that end, the PICO question was formulated according to which the aim of this study was to review the available evidence on the association between therapy-related anticholinergic burden—or failing that, the use of drugs with anticholinergic activity—and the onset of xerostomia or xerophthalmia in patients older than 65 years.

## Method

A systematic review was conducted following the general principles of the Centre for Reviews and Dissemination (CRD) [20] and the recommendations of the PRISMA (Preferred Reporting Items for Systematic Reviews and Meta-Analyses) Statement [21].

### Search strategy and data sources

The search strategy was developed based on the PICO question, until August 2022, in three databases: PUBMED, CINAHL and EMBASE. A combination of MeSH terms and key words was used (Table 1).

**Table 1:** Search strategy

PUBMED	((((elderly) OR (aged [Mesh Term]) OR (older))) AND (((anticholinergic drugs) OR muscarinic antagonist [Mesh Term]) OR “agents, anticholinergic”) OR anticholinergic load) OR anticholinergic burden) OR ACh scales)) AND ((((xerophthalmia [Mesh Term]) OR dry eye)) OR ((dryness, mouth OR xerostomia [Mesh Term] OR hyposalivation OR dry mouth) OR (“negative outcomes OR “anticholinergic side-effects”))). Filter: (SPECIES: “humans”; AGE: “Aged: 65+ years”; LANGUAGE: “English”, “Spanish”).
CINAHL	(‘cholinergic receptor blocking agent’ OR ‘muscarinic receptor blocking agent’ OR ‘burden anticholinergic’ OR ‘anticholinergic load’ OR ‘ACh Scales’) AND (‘xerostomia’ OR ‘hyposalivation’ OR ‘xerophthalmia’ OR ‘eye dry’) AND ([aged]/lim OR [very elderly]/lim) LANGUAGE: “English”, “Spanish”
EMBASE	(‘cholinergic receptor blocking agent’ OR ‘muscarinic receptor blocking agent’ OR ‘burden anticholinergic’ OR ‘anticholinergic load’ OR ‘ACh scales’) AND (‘xerostomia’ OR ‘dry mouth’ OR ‘hyposalivation’ OR ‘xerophthalmia’ OR ‘eye dry’) AND ([aged]/lim OR [very elderly]/lim) LANGUAGE: “English”, “Spanish”

#### Selection criteria

Inclusion criteria included observational or experimental studies relating anticholinergic activity (i.e., anticholinergic risk or burden) or failing that, anticholinergic therapy, to adverse effects like xerostomia or xerophthalmia in people aged 65 or older in any clinical or social context.

Letters to the editor, dissertations, clinical cases, case series and conference abstracts were excluded, as were studies published in a language other than Spanish or English.

#### Study identification and data extraction

Studies were selected by two reviewers (PME and CGP). First, after duplicates were removed, references were screened for inclusion based on their title and abstract. In order to enhance consistency, both reviewers evaluated the first 50 references. Subsequently, full-text articles were reviewed. Discrepancies were resolved by consensus between two external reviewers (RRH and SFS). The reasons for exclusion of full-text articles are detailed in Appendix 1.

The following variables were extracted from each study:

- Study-related variables: study design, duration (months), country and sample size.
- Population-related variables: demographic variables (age and sex) and type of population studied.
- Variables related to anticholinergic activity: anticholinergic activity (use of anticholinergics or measurement of anticholinergic burden), burden measurement method (anticholinergic scale used), anticholinergic exposure (proportion of the population exposed to anticholinergics) and most frequently prescribed anticholinergics (most frequent active substances or drug classes).
- Variables related to anticholinergic peripheral adverse effects (xerostomia or xerophthalmia): prevalence of xerostomia or xerophthalmia, and assessment methods used.
- Outcome-related variables: analysed association (between anticholinergic burden or the use of anticholinergics and peripheral adverse effects), type of association ((+) existent or (−) non-existent), association measurement and magnitude of each association variable.

Other potential causes of xerostomia and xerophthalmia were also taken into consideration as confounding factors, based on their likelihood of inclusion in the articles selected:

Xerostomia: diabetes, rheumatoid arthritis, Sjögren syndrome, polypharmacy and radiotherapy.

Xerophthalmia: diabetes, rheumatoid arthritis, Sjögren syndrome, polypharmacy and eye surgery.

### Quality of articles

Different tools were used to assess the internal validity of the included studies depending on the study design. Cross-sectional studies were assessed using the Joanna Briggs Institute (JBI) critical appraisal checklist [22], the Newcastle-Ottawa Scale (NOS) [23] was used to assess cohort and case-control studies, and the Cochrane risk-of-bias tool for randomized trials (Rob 2) [24].

## Results

**Fig. 1:**
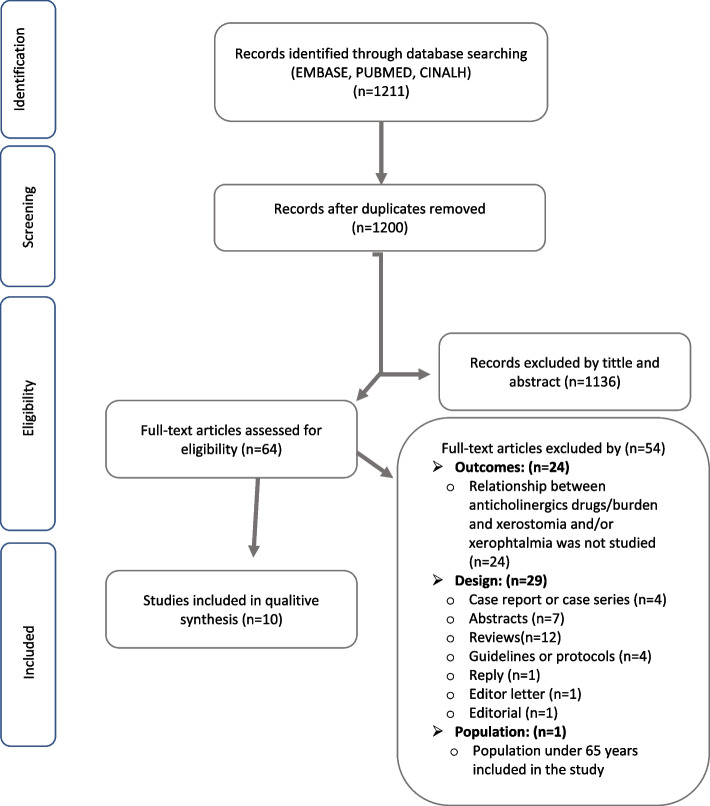
Flow chart of the study selection

The search strategy applied to the three databases selected resulted in 1211 studies, out of which 11 duplicates were removed. A total of 1136 studies were excluded based on their title and abstract, while 64 studies were pre-selected, out of which 10 were finally included in the review (Fig. 1): six descriptive studies, [25–30] three analytical observational studies [31–33] and one clinical trial [34].

The article description differentiates the articles that relate the use of anticholinergics to adverse effects (xerostomia or xerophthalmia) [27–29, 31] without measuring the anticholinergic burden, from those that analyze this association by relating the anticholinergic burden to the drug therapy of each patient [25, 26, 30, 32–34].

### Population profile

The profile of the elderly population studied in each article is shown in Table 2. Collectively, the 10 studies included in the review involved 3622 patients with an average age of over 75 years. Social and clinical contexts were diverse, with patients living alone, [25, 26, 31, 32] in care homes, [27, 28] as well as admitted to nursing homes and acute hospital wards [29, 30, 33]. Nearly all of them have an observational design and were conducted in high-income Western countries.

**Table 2:** General characteristics of the included studies

	Study Year	Study design	Population	Country	Sex (female %)	Age (y)	N	Duration (M^**a**^)
Anticholinergicdrug use and peripheral effects	Thomson WM. 1993	Cross-sectional study	Nursing home residents and geriatric hospital residents	New Zealand	60%	82.5 ± 2.4^𝜹^	359	–
	Katz IR. 1988	Cross-sectional study	Congregate Housing population	United States	33%	83.2 ± 5.8^𝜹^	53	–
	Ness J. 2006	Prospective cohort study	Community-dwelling patients	United States	2.1%	74.3	532	3
	Desoutter A. 2012	Cross-sectional study	Long term geriatric ward	France	no data	84.6 ± 8.4^𝜹^	769	–
Anticholinergic burden and peripheral effects	Rudolph JL. 2008	Retrospective Cohort study	Community-dwelling patients	United States	3%	78.7 ± 5.3^𝜹^	132	9
		Prospective Cohort study			0%	71.5 ± 11.6^𝜹^	117	10
	Kersten H. 2012	Cross-sectional study	Nursing home residents	Norway	79.3%	> 73	87	–
	Kersten H. 2013	Randomized Controlled Trial	Nursing home residents	Norway	79.3%	> 73	87	2
	Tiisanoja A. 2017	Cross-sectional study	Community-dwelling patients	Finland	73.6%	79.6 ± 3.7^𝜹^	152	–
	Inkeri NM. 2019	Cross-sectional study	Community-dwelling patients	Finland	50.9%	> 65	1.084	–
	Lavrador M. 2021	Case control Study	Patients admitted in Internal Medicine wards	Portugal	50	81.7 ± 7.8 ^𝜹^	250	12

^𝜹^ Average (Standard deviation); ^a^ M: months

### Measurement of anticholinergic activity and peripheral adverse effects: xerophthalmia and xerostomia

Out of all the articles included, six described xerostomia and four reported both adverse effects. Seven articles provided prevalence data, with xerostomia at 10.6–59.2% [26–29, 31–33]. However, only three measured xerophthalmia, with a prevalence range of 5.1–34% [31–33] (Table 3).

**Table 3:** Xerostomia and xerophthalmia prevalence, assessment methods and others related variables

Relation studied	Study. year	Xerostomia		Xerophthalmia		Anticholinergic Drug	
		Assessment methods	Prevalence (%)	Assessment methods	Prevalence (%)	Most frequently prescribed anticholinergic drugs	Exposure (%)
Anticholinergic drug use and peripheral effects	Thomson WM. 1993	Patient’s self-reports	20%	No tested	No tested	No data	3.9%
	Katz IR. 1988	Patient’s self-reports	39%	No tested	No tested	Antihistamines	24.5%
	Ness J. 2006	Patient’s self-reports	51.60%	Patient’s self-reports and eye tears prescription	9.90%	Antihistamines	27.1%
	Desoutter A. 2012	Patient’s self-reports	37.30%	No tested	No tested	No Data	27.8%
Anticholinergic burden and peripheral effects	Rudolph JL.2008	Retrospective cohort: documented in the medical record	10.60%	Retrospective cohort: documented in the medical record	13.6%	No Data	46.9%^a^
		Prospective cohort: Patient’s self-reports	44.40%	Prospective cohort: Patient’s self-reports	5.1%	No Data	29.9%^a^
	Kersten H. 2012	Clinical assesment: ST^c^	No Data	No tested	No tested	Antidepressants	100%^b^
	Kersten H. 2013	Clinical assesment: ST^c^	No Data	No tested	No tested	Furosemide	100%^b^
	Tiisanoja A. 2017	Clinical assesment: SSD^d^	21.40%	No tested	No tested	No Data	49%^a^
	Inkeri NM. 2019	Patient’s self-reports	No Data	Patient’s self-reports	No Data	Antihistamines	8.9%^a^
	Lavrador M. 2021	Clinical assesment: ST^c^	59.2%	Clinical assesment: SchT^e^	34%	No data	68%^f^ 83.6%^b^ 34.8%^a^ 78%^g^

^a^Anticholinergic exposure by ARS scale; ^b^ Anticholinergic exposure by ADS scale; ^c^ST: Swab technique; ^d^ SSD: Salivary Secretion Draining; ^e^ SchT: Schirmer Test; ^f^Anticholinergic exposure by DBI score; ^g^ Anticholinergic exposure by ACB

In order to assess xerostomia, five studies used self-administered questionnaires, [27–29, 31, 32] three used clinical tests [30, 33, 34] and one combined questionnaires with clinical tests [26] (Appendix 3). In the case of xerophthalmia, three studies used self-administered questionnaires [25, 31, 32] and one used a clinical test [33].

With respect to the exposure of the population to anticholinergics, it was highly variable, ranging from 3.9 to 100%. Only five studies [25, 28, 30, 31, 34] report data about the most prevalent drug class or active substance with anticholinergic properties among the study population. Antihistamines are the most prevalent drugs [25, 28, 31] in three of the five articles providing data.

Out of the five studies that measured the burden, three used the ARS scale, [25, 26, 32] two used the ADS, [30, 34] and one used the DBI (Drug Burden Index) [35] and ACB (Anticholinergic Cognitive Burden Scale) [36] in addition to ARS and ADS [33].

#### Xerostomia and xerophthalmia related to the use of anticholinergics

Four studies found a statistically significant association between the use of anticholinergics and the prevalence of xerostomia [28, 30, 33, 34]. Only one study [27] measured the association between xerophthalmia and the use of anticholinergics, which was found non-existent (Table 4).

**Table 4:** Association between anticholinergic burden or anticholinergic use and xerostomia and xerophthalmia

Relation studied	Study. year	Scale	Peripherical adverse effect	Patient’s groups compared when looking for a correlation	Association	Association magnitude
Anticholinergic drug use and peripheral effects	Thomson WM. 1993	No Scale	Xerostomia	Occurrence of reported dry mouth in patients taking anticholinergics drugs vs patient not taken	**+**	50% vs 19% (*p* = 0.02) ^b^
	Katz IR. 1988	No Scale	Xerostomia	Dry mouth reported by patients that taking anticholinergics drugs (PNS + CNS) vs patients not taking anticholinergic drugs	**+**	69% vs 29% (*p* < 0.05) ^c^
			Xerostomia	Dry mouth reported by patients that taking anticholinergics drugs (CNS) vs patients not taking anticholinergic drugs	**+**	64% vs 29% (p < 0.05) ^c^
	Ness J. 2006	No Scale	Xerostomia	Used at least 1 anticholinergic agent vs did not use any anticholinergic	**+**	−0.7((−1.1)-0.3) (*p* < 0.01)^d^
			Xerophthalmia		**–**	−0.2 ((−0.3)-(−0.1) (*p* = 0.39)^d^
	Desoutter A. 2011	No Scale	Xerostomia	Patients with anticholinergics drug use vs patients without anticholinergic drugs	**+**	1.35 (1.05–1.73) (p = 0.02)^e^
Anticholinergic burden and peripheral effects	Rudolph JL. 2008	ARS	Peripheral adverse events^a^	Retrospective cohort: ARS = 0, ARS = 1–2 vs ARS ≥ 3	**+**	1.4 (1.0–1.9)^f^
				Prospective cohort ARS = 0,ARS = 1–2 vs ARS ≥ 3	**+**	2.1 (1.5–2.9)^f^
	Kersten H. 2012	ADS	Xerostomia	ADS = 3 vs ADS = 4	**–**	0.0 (−0.3-(0.3))^g^
				ADS = 3 vs ADS = 5	**–**	− 0.1 (− 0.3-(0.2))^g^
				ADS = 3 vs ADS≥6	**+**	−0.4 (− 0.7-(0.1)) (*p* < 0.001)^g^
	Kersten H. 2013	ADS	Xerostomia	Intervention group vs control group	**–**	−0.07 (− 0.21–0.07) (*p* = 0.34)^h^
	Tiisanoja A. 2017	ADS	Salivary estimulated secretion	ADS = 0 vs ADS≥3	**–**	1.50 (0.80–2.81) ^i^
			Salivary unestimulated secretion	ADS = 0 vs ADS≥3	**+**	2.31 (1.22–4.43)^i^
			Xerostomia	ADS = 0 vs ADS≥3	**+**	3.17 (1.44–6.96)^i^
	Inkeri NM. 2019	ARS	Xerophthalmia	ARS = 0 vs ARS > 0	**–**	No numerical data available
				Diabetic patients with ARS = 0 vs diabetic patients with ARS > 0	**–**	
			Xerostomia	ARS = 0 vs ARS > 0	**–**	No numerical data available
				Diabetic patients with ARS = 0 vs diabetic patients with ARS > 0	**–**	
	Lavrador M. 2021	ARS	Xerophthalmia	Differences between the median of anticholinergic burden scale scores in patients with xerostomia or xeropthalmia vs the median of anticholinergic burden scale scores in patients without xerostomia or xeropthalmia	**+**	1.0 vs 0.0 (*p* = 0.001)^j^
			Xerostomia		**+**	1.0 vs 0.0 (p < 0.001)^j^
		ADS	Xerophthalmia		**+**	2.0 vs 2.0 (*p* = 0.020)^j^
			Xerostomia		**+**	2.0 vs 1.5 (p < 0.001)^j^
		ACB	Xerophthalmia		**+**	3.0 vs 1.0 (p < 0.001)^j^
			Xerostomia		**+**	2.0 vs 1.0 (p < 0.001)^j^
		DBI	Xerophthalmia		**+**	0.96 vs 0.50 (p < 0.001)^j^
			Xerostomia		**+**	0.75 vs 0.13 (p < 0.001)^j^

^a^Peripherical adverse effects: adverse effects include dry mouth, dry eyes and constipation. ^b^Chi square test; ^c^ Pearson χ2; ^d^Analysis of variance for continuous variables; ^e^Odd Ratio, predictors of xerostomia identified by multivariable analysis; ^f^Relative Risk adjusted for age and number of medications; ^g^Analysis of covariance performed with log-transformed data; ^h^Analysis of covariance between the groups analysed; ^I^Relative Risk adjusted by age, gender, education, diabetes and rheumatoid disease, and the Functional Comorbidity Index by a Poisson regression model; ^j^Mann-Whitney test

ACB: Anticholinergic Cognitive Burden Scale; ADS: Anticholinergic Drug Scale; ARS: Anticholinergic Risk Scale; CNS: central nervous system; DBI: Drug Burden Index; PNS: peripheral nervous system;

#### Anticholinergic burden-related xerostomia and xerophthalmia

Several studies found a significant connection (Table 4) between the presence of xerostomia or xerophthalmia and the level of anticholinergic burden. Rudolph JL et al. found a positive association [32] but considered a compound variable (peripheral effects) including xerostomia, xerophthalmia and constipation.

A positive association was found when comparing the prevalence of xerostomia in patients with a high anticholinergic burden versus patients with lower [30] or no [26] anticholinergic burden.

Xerostomia and xerophthalmia were more prevalent in patients with significantly higher levels of anticholinergic burden [33].

No association was found between the prevalence of xerostomia or xerophthalmia when comparing patients with any level of anticholinergic burden—that is, not stratifying them by level of burden—versus no burden [25].

Out of all studies, only one clinical trial, considering an interventional reduction of the median ADS score by 2 units, [34] did not show any difference in the salivary secretion 4 weeks after the intervention. Both study groups had similar salivary flow rates.

### Confounding factors

All studies, except for one, considered polypharmacy or the number of prescribed drugs as a variable [28]. The mean or median along with the standard deviation or range are the most frequently collected data.

Three studies included diabetes and rheumatoid arthritis in the population comorbidity assessment through the Functional Comorbidity Index or the Charlson Comorbidity Index, [30, 33, 34] while one other study considered these factors as individual variables, excluding them from the comorbidity scale used [26].

All studies used a multivariable analysis adjusted to these co-variables in their statistical analysis.

None of the studies included measured head and neck radiotherapy in xerostomia or eye surgery in xerophthalmia as variables, nor Sjögren syndrome in xerostomia or xerophthalmia (Supplementary Table S1; Supplementary Table S2).

### Quality of articles

Three different types of tools were used to assess studies quality (Appendix 2), JBI [22] for cross-sectional studies, [25–30] NOS [23] for cohort studies [31, 32] and case-control study [33] and finally. The clinical trial [34] was assessed through the Cochrane Risk of Bias tool (Rob 2) [24]. Two cross-sectional studies were considered methodologically adequate [26, 30], ensuring at least 7 of the 8 requirements established by the JBI were met. The rest of the observational studies presented a risk of bias in their results.

The use of non-validated tools for measuring the variables studied, as well as the lack of control of confounding factors in the population studied, were the main problems of internal validity [25, 27–29].

Regarding the cohort studies [31, 32], a NOS score of 6 and 5 stars, respectively, was obtained and a NOS score of 6 for the case-control study [33]. It should be noted that the maximum score is 9 stars for each type of study.

For lastly the clinical trial was qualified as a low risk of bias by rob 2.0 tool.

## Discussion

This systematic review consisted in a comprehensive literature search aimed at selecting studies that assessed the peripheral effects (xerostomia and xerophthalmia) associated with the use of medications with anticholinergic activity. Despite the prevalence of such problems and the importance of their direct and indirect effects, very few papers were found to assess them.

The findings of this review help understand the current state of the issue and move forward in the study of anticholinergic-related peripheral effects. The methods used in the studies included are fundamentally observational but very heterogeneous, and there is high variability among the tools used to obtain the data and measure the effects.

To determine if patients presented with xerostomia, some studies used self-administered questionnaires [25, 27–29, 31, 32] while others used clinical tests, [30, 33, 34] including one that complemented clinical tests with questionnaires [26]. Given the method variability across studies, outcomes may differ significantly depending on the tools used.

With respect to xerostomia, it should be made clear that it is not necessarily related to hyposalivation. This matter is addressed by Villa et al. [37] in their review on the diagnosis of xerostomia and hyposalivation, concluding that the determination of the salivary flow rate through a clinical test is essential to accurately make a connection between xerostomia and hyposalivation. Similarly, where hyposalivation is diagnosed, xerostomia must be confirmed through validated questionnaires, as not all patients with hyposalivation report xerostomia.

Within this review, out of seven studies using questionnaires to assess xerostomia, at least four use different questionnaires [25–27, 29, 31] and two do not detail the questionnaire used, only stating that patients are questioned to determine if they have xerostomia [28, 32]. The use of validated tools like Xerostomia Inventory, [38] which even offers cross-cultural adaptations [12] to different languages, would have made the outcomes more valid.

In this review, polypharmacy, diabetes and rheumatoid arthritis were considered as the main confounding variables for xerostomia and xerophthalmia, versus anticholinergic burden or the use of drugs with anticholinergic activity. It is worth stressing that the Sjögren syndrome, [39] characterised by xerostomia/hyposalivation and xerophthalmia, is not explicitly mentioned in the exclusion criteria or as an adjustable variable in the articles. This could be justified by its low prevalence, underdiagnosis or close connection with rheumatoid arthritis. However, a number of articles [27, 29, 31, 32] do not consider any confounding factor, which leads to unadjusted data and therefore, data at risk of bias.

The scales applied in these studies were the ADS, [40] ARS, [32] ACB [36] and DBI [35]. The DBI scale differs from the rest of the tools in considering the dosage of anticholinergics and/or sedatives. In general, there is high variability among anticholinergic scales, [41] probably due to the differences in the drugs included. The ARS includes only 49 drugs while the ADS includes 117. Many of the drugs included in the ADS scale are therefore overlooked by the ARS, which could mean that the level of anticholinergic burden may also vary within the same population. In other hand, the scores given to the drugs are different between scales. This may happen because the development methods and validation studies are different across the scales. Also, many of them are based on consensus techniques highly dependent on expert panels’ knowledge. This variability is shown in the 250-patient study conducted by Lavrador M, [33] where the proportion of patients taking at least one drug with anticholinergic activity varied significantly depending on the scale used.

With respect to the potential association between anticholinergic burden and the onset of xerostomia or xerophthalmia, Rudolph J et al. [32] found a link between a high anticholinergic burden (ARS = 3) and the presence of peripheral adverse effects. However, this study used a compound variable (peripheral effects) including xerostomia, xerophthalmia and constipation, and therefore cannot be used to estimate each magnitude separately or the specific relationship between anticholinergic burden and xerostomia or xerophthalmia. It appears that the connection between xerostomia and anticholinergic burden is close when patients present with high levels of anticholinergic burden [26, 30]. In patients with low or intermediate levels of anticholinergic burden, this association is less conclusive. Future studies could attempt to establish some anticholinergic burden ranges in order to identify the threshold of association with the peripheral effects analysed. This would allow researchers to identify the level of anticholinergic burden that must be reduced to obtain clinical benefit, as well as to standardise the use of scales considering the drug dosage—e.g., the DBI, which may allow for a more accurate anticholinergic burden measurement, as it considers dose reduction in cases where there is no therapeutic alternative or it is not possible to deprescribe the anticholinergic in question. In fact, the impossibility of deprescription could be one of the reasons why in Kersten H et al., [34] the burden reduction intervention did not improve salivary secretion.

The relationship between xerophthalmia and anticholinergic burden or the use of anticholinergics was inconclusive due to the limited number of studies assessing such adverse effect.

One strength of this literature review is its extensiveness, as it was not restricted by a publication deadline and included all relevant studies on the association between peripheral effects (xerophthalmia and xerostomia), and both anticholinergic burden and the use of anticholinergics. No other review was found to analyse the association between these peripheral effects and the anticholinergic burden or the use of anticholinergics, so it can be stated that this is the first review to do so. One of the main constraints was the limited number of studies meeting the inclusion criteria and, more specifically, analysing xerophthalmia. Moreover, most studies included were observational, only one of them being a clinical trial, which decreased the quality of the evidence and limited the conclusions from the findings.

## Conclusion

There are very few data relating anticholinergic burden or the use of anticholinergics to xerophthalmia. However, there seems to be a closer connection between a high anticholinergic burden, as well as the use of anticholinergics, and the occurrence of xerostomia. Further studies that properly characterise the study population and use measurement variables based on validated tools may be necessary to standardise the outcomes and thus make them more easily comparable.

## Supplementary Information

**Additional file 1:** **Appendix 1.** Full text articles excluded and causes of exclusion.

**Additional file 2:** **Appendix 2.** Quality assessment of included studies (JBI Critical Appraisal tool for Cross-sectional studies, Newcastle-Ottawa Quality Assessment Scale for cohort and case-control studies, Rob 2.0 tool for randomized trials).

**Additional file 3:** **Appendix 3.** Tools used for measure Xerostomia and Xerophthalmia (Description of the methods used in the studies to measure xerostomia and/or xerophthalmia).

**Additional file 4:** **Supplementary table S1.** Alternative causes of Xerophthalmia (Confounding factors for Xerophthalmia considered in the studies).

**Additional file 5:** **Supplementary table S2.** Alternative causes of Xerostomia (Confounding factors for Xerostomia considered in the studies).
