# Supplementary material for: Association between anticholinergic activity and xerostomia and/ or xerophthalmia in the elderly: systematic review
Source: BMC Pharmacol Toxicol. 2022 Dec 21;23:94. doi: 10.1186/s40360-022-00637-8 (PMC9769019; doi:10.1186/s40360-022-00637-8)
Supplement: Supplementary file 1 — Additional file 1: Appendix 1. Full text articles excluded and causes of exclusion. [file 40360_2022_637_MOESM1_ESM.docx]

**Association between anticholinergic activity with xerostomia and/or xerophthalmia in elderly: a systematic review**

**Authors:**

Prado-Mel E^1^, Ciudad-Gutiérrez P^1^, Rodríguez-Ramallo H^1^, Sánchez-Fidalgo S^2^, Santos-Ramos B^1^, Villalba-Moreno AM^1^

1. Hospital Universitario Vírgen del Rocío, (Pharmacy department), Seville, (Andalucía), Spain.

2. University of Seville, (Department of Preventive Medicine and Public Health), Seville, (Andalucía), Spain.

**Corresponding author**: Sánchez-Fidalgo S, Avenida Dr Fedriani SN, Sevilla, CP: 41009, telephone: 954551771; [fidalgo@us.es](mailto:fidalgo@us.es)

ORCID 0000-0002-3630-7122

**Appendix 1. Full text articles excluded and causes of exclusion.**

| **Reference** | **Exclusion criteria** |
| --- | --- |
| Lloyd PM. Xerostomia: not a phenomenon of aging. Wis Med J. 1983;82(9):21-2. | Narrative Review |
| Baker KA, Ettinger RL. Intra-oral effects of drugs in elderly persons. Gerodontics. 1985;1(3):111-6. | Narrative Review |
| Ben-Aryeh H, Miron D, Berdicevsky I, Szargel R, Gutman D. Xerostomia in the elderly: prevalence, diagnosis, complications and treatment. Gerodontology. 1985;4(2):77-82. | No association assessment between anticholinergic effect (burden/use) with xerostomia and/or xerophthalmia |
| Närhi TO, Meurman JH, Ainamo A, Nevalainen JM, Schmidt-Kaunisaho KG, Siukosaari P, Valvanne J, Erkinjuntti T, Tilvis R, Mäkilä E. Association between salivary flow rate and the use of systemic medication among 76-, 81-, and 86-year-old inhabitants in Helsinki, Finland. J Dent Res. 1992;71(12):1875-80. | No association assessment between anticholinergic effect (burden/use) with xerostomia and/or xerophthalmia. |
| Hayasbi T, Yamawaki S, Nisbikawa T, Jeste DV. Usage and Side Effects of Neuroleptics in Elderly Japanese Patients. Am J Geriatr Psychiatry. 1995;3(4):308-316. | No association assessment between anticholinergic effect (burden/use) with xerostomia and/or xerophthalmia |
| Fastbom J, Claesson CB, Cornelius C, Thorslund M, Winblad B. The use of medicines with anticholinergic effects in older people: a population study in an urban area of Sweden. J Am Geriatr Soc. 1995;43(10):1135-40. | No association assessment between anticholinergic effect (burden/use) with xerostomia and/or xerophthalmia |
| Astor FC, Hanft KL, Ciocon JO. Xerostomia: a prevalent condition in the elderly. Ear Nose Throat J. 1999;78(7):476-9. | Case Report |
| Ghezzi EM, Wagner-Lange LA, Schork MA, Metter EJ, Baum BJ, Streckfus CF, Ship JA. Longitudinal influence of age, menopause, hormone replacement therapy, and other medications on parotid flow rates in healthy women. J Gerontol A Biol Sci Med Sci. 2000;55(1):M34-42. | No association assessment between anticholinergic effect (burden/use) with xerostomia and/or xerophthalmia |
| Mintzer J, Burns A. Anticholinergic side-effects of drugs in elderly people. J R Soc Med. 2000;93(9):457-62. | Narrative Review |
| Oral dryness adds to problems in the elderly. Drugs Ther Perspect [Internet]. 2000 [Cited 2020 Mar 15];16(4):12-5. Available from : https://link.springer.com/article/10.2165%2F00042310-200016040-00005 | Guideline |
| Patel PS, Ghezzi EM, Ship JA. Xerostomic complaints induced by an anti-sialogogue in healthy young vs. older adults. Spec Care Dentist. 2001;21(5):176-81. | No association assessment between anticholinergic effect (burden/use) with xerostomia and/or xerophthalmia |
| Tune LE. Anticholinergic effects of medication in elderly patients. J Clin Psychiatry. 2001;62 Suppl 21:11-4. | Narrative Review |
| Miller CA. Anticholinergics: the good and the bad. Geriatr Nurs. 2002;23(5):286-7. | Guideline |
| Zinner NR, Mattiasson A, Stanton SL. Efficacy, safety, and tolerability of extended-release once-daily tolterodine treatment for overactive bladder in older versus younger patients. J Am Geriatr Soc. 2002;50(5):799-807. | No association assessment between anticholinergic effect (burden/use) wit  h xerostomia and/or xerophthalmia |
| Vangala VR, Tueth MJ. Chronic anticholinergic toxicity. Identification and management in older patients. Geriatrics. 2003;58(7):36-7. | Case Report |
| Kay GG, Granville LJ. Antimuscarinic agents: implications and concerns in the management of overactive bladder in the elderly. Clin Ther. 2005;27(1):127-38. | Narrative Review |
| Swagerty D, Brickley R; American Medical Directors Association; American Society of Consultant Pharmacists. American Medical Directors Association and American Society of Consultant Pharmacists joint position statement on the Beers List of Potentially Inappropriate Medications in Older Adults. J Am Med Dir Assoc. 2005;6(1):80-6. | Guideline |
| Bellia V, Battaglia S, Matera MG, Cazzola M. The use of bronchodilators in the treatment of airway obstruction in elderly patients. Pulm Pharmacol Ther. 2006;19(5):311-9. | Narrative Review |
| Cefalu, C.A.Drug therapy in elderly patients: How to avoid adverse effects and interactions. Consultant. 2006; 46(14):1545-52. | Narrative Review |
| Han L, Agostini JV, Allore HG. Cumulative anticholinergic exposure is associated with poor memory and executive function in older men. J Am Geriatr Soc. 2008;56(12):2203-10. | No association assessment between anticholinergic effect (burden/use) with xerostomia and/or xerophthalmia |
| Gallagher L, Naidoo P. Prescription drugs and their effects on swallowing. Dysphagia. 2009;24(2):159-66. | No association assessment between anticholinergic effect (burden/use) with xerostomia and/or xerophthalmia |
| Porter SR. Plenary abstract: Xerostomia: prevalence, assessment, differential diagnosis and implications for quality of life. Oral Dis. 2010;16(6):501-2. | Narrative Review |
| Carnahan RM, Lund BC, Chrischilles EA, Kaboli PJ. Relation of symptom experience with anticholinergic drug exposure in older veterans. J Pharm Pract . 2010;23(2):159. | Abstract |
| Boparai MK, Korc-Grodzicki B. Prescribing for older adults. Mt Sinai J Med. 2011;78(4):613-26. | Narrative Review |
| Sprung, DJ. Do Anticholinergic Medications Really Pose a Higher Risk for Adverse Effects in Irritable Bowel Syndrome Patients Over Age 65? A Community Based Study. Gastroenterol. 2011; 140(5), S–359*.* | Abstract |
| Lowry E, Woodman RJ, Soiza RL, Mangoni AA. Associations between the anticholinergic risk scale score and physical function: potential implications for adverse outcomes in older hospitalized patients. J Am Med Dir Assoc. 2011;12(8):565-72. | No association assessment between anticholinergic effect (burden/use) with xerostomia and/or xerophthalmia |
| Uusvaara J, Pitkala KH, Kautiainen H, Tilvis RS, Strandberg TE. Association of anticholinergic drugs with hospitalization and mortality among older cardiovascular patients: A prospective study. Drugs Aging. 2011;28(2):131-8. | Narrative Review |
| Güneş Z, Denat Y, Müezzinoğlu M, Sen S, Yılmaz S, Atlı E. The risk factors effecting the dry mouth in inpatients in Hospital in west Anatolia. J Clin Nurs. 2012;21(3-4):408-14. | No association assessment between anticholinergic effect (burden/use) with xerostomia and/or xerophthalmia |
| Karimi S, Dharia SP, Flora DS, Slattum PW. Anticholinergic burden: clinical implications for seniors and strategies for clinicians. Consult Pharm. 2012;27(8):564-82. | Narrative Review |
| Ben-Omar N,  Wenzel-Seifert K, Haen E. Age-dependency of the risk for tricyclic antidepressant-associated anticholinergic adverse reactions. Pharmacopsichiatry. 2012; 45(6):252-A4 | Abstract |
| Koshoedo S, Soiza RL, Purkayastha R, Mangoni AA. Anticholinergic drugs and functional outcomes in older patients undergoing orthopaedic rehabilitation. Am J Geriatr Pharmacother. 2012;10(4):251-7. | No association assessment between anticholinergic effect (burden/use) with xerostomia and/or xerophthalmia |
| Ben-Omar N, Wenzel-Seifert K, Hefner G, Hiemke C, Haen E. Age-dependency of psychotropic drug-induced anticholinergic adverse events: Analysis of the data bank of the pharmacovigilance system AGATE. Naunyn Schmiedebergs Arch Pharmacol. 2013;386:S90. | Abstract |
| Kersten H, Wyller TB, Molden E. Association between inherited CYP2D6/2C19 phenotypes and anticholinergic measures in elderly patients using anticholinergic drugs. Ther Drug Monit. 2014 ;36(1):125-30. | No association assessment between anticholinergic effect (burden/use) with xerostomia and/or xerophthalmia |
| Gray SL, Anderson ML, Dublin S, Hanlon JT, Hubbard R, Walker R, Yu O, Crane PK, Larson EB. Cumulative use of strong anticholinergics and incident dementia: a prospective cohort study. JAMA Intern Med. 2015;175(3):401-7. | No association assessment between anticholinergic effect (burden/use) with xerostomia and/or xerophthalmia |
| Griebling TL. Re: Anticholinergic Drug Use and Negative Outcomes among the Frail Elderly Population Living in a Nursing Home. J Urol. 2015;194(2):477. | Reply |
| Joyau C, Veyrac G, Delamarre D F, Pasquier A, Priez J, Jolliet P. Drug-induced taste disorders: Prescribing analysis in French nursing homes. Eur Geriatr Med [Internet]. 2015 [Cited 2020 Abr 15]. Available from: https://www.sciencedirect.com/science/article/abs/pii/S1878764915305040 | Abstract |
| Yayla EM, Yavuz E, Bilge U, Keskin A, Binen E. Drugs with anticholinergic side-effects in primary care. Niger J Clin Pract. 2015;18(1):18-21. | No association assessment between anticholinergic effect (burden/use) with xerostomia and/or xerophthalmia |
| Collamati A, Martone AM, Poscia A, Brandi V, Celi M, Marzetti E, Cherubini A, Landi F. Anticholinergic drugs and negative outcomes in the older population: from biological plausibility to clinical evidence. Aging Clin Exp Res. 2016;28(1):25-35. | Narrative Review |
| Bostock C, McDonald C. Antimuscarinics in Older People: Dry Mouth and Beyond. Dent Update. 2016;43(2):186-8, 191. | Case Report |
| Kachru N, Sura S, Chatterjee S, Aparasu RR. Antimuscarinic Medication Use in Elderly Patients with Overactive Bladder. Drugs Aging. 2016;33(10):755-763. | Abstract |
| Lampela P, Taipale H, Hartikainen S. Association Between Anticholinergic Load and Frailty in Community-Dwelling Older People. J Am Geriatr Soc. 2016;64(3):671-2. | Editor Letter |
| Ozen Tunay Z, Ozdemir O, Ergintürk Acar D, Cavkaytar S, Ersoy E. Dry eye findings worsen with anticholinergic therapy in patients with urge incontinence. Int Urogynecol J. 2016;27(6):919-22. | No association assessment between anticholinergic effect (burden/use) with xerostomia and/or xerophthalmia |
| Salahudeen MS, Nishtala PS, Duffull SB. The Influence of Patient Characteristics on Anticholinergic Events in Older People. Dement Geriatr Cogn Dis Extra. 2016;5(3):530-41. | No association assessment between anticholinergic effect (burden/use) with xerostomia and/or xerophthalmia |
| Brown RS, Rhodes BH, Siewe MS, Matthews TJ. Severe Xerostomia Secondary to Anticholinergic Drug Therapy: Case Report. Dent Today. 2017 Feb;36(2):136-8. | Case Report |
| Lee KC, Seong BM. Does Systemic Disease Aggravate the Severity of Dry Mouth by Anticholinergics in Overactive Bladder Patients? Urol J. 2017;14(2):3035-3039. | No association assessment between anticholinergic effect (burden/use) with xerostomia and/or xerophthalmia |
| Schoenmakers TW, Teichert M, Wensing M, de Smet PA. Evaluation of Potentially Drug-Related Patient-Reported Common Symptoms Assessed During Clinical Medication Reviews: A Cross-Sectional Observational Study. Drug Saf. 2017;40(5):419-430. | No association assessment between anticholinergic effect (burden/use) with xerostomia and/or xerophthalmia |
| Eibling D. Frailty and Polypharmacy in Older Patients with Otolaryngologic Diseases. Clin Geriatr Med. 2018;34(2):289-298. | Guideline |
| Ivchenko A, Bödeker RH, Neumeister C, Wiedemann A. Anticholinergic burden and comorbidities in patients attending treatment with trospium chloride for overactive bladder in a real-life setting: results of a prospective non-interventional study. BMC Urol. 2018;18(1):80. | No association assessment between anticholinergic effect (burden/use) with xerostomia and/or xerophthalmia |
| Kiesel EK, Hopf YM, Drey M. An anticholinergic burden score for German prescribers: score development. BMC Geriatr. 2018 11;18(1):239. | No association assessment between anticholinergic effect (burden/use) with xerostomia and/or xerophthalmia |
| Rhee TG, Choi YC, Ouellet GM, Ross JS. National Prescribing Trends for High-Risk Anticholinergic Medications in Older Adults. J Am Geriatr Soc. 2018;66(7):1382-1387. | No association assessment between anticholinergic effect (burden/use) with xerostomia and/or xerophthalmia |
| Lavrador M, Bento C, Caramona MM, Fernandez-Llimos F, Figueiredo IV, Castel-Branco MM. Anticholinergic burden versus anticholinergic effects in institutionalized elderly. Int J Clin Pharm. 2019;41(2):598. | Abstract |
| Amoros-Reboredo P, Soy D, Hernandez-Hernandez M, Lens S, Mestres C. Anticholinergic Burden and Safety Outcomes in Older Patients with Chronic Hepatitis C: A Retrospective Cohort Study. Int J Environ Res Public Health. 2020;17(11):3776. | No association assessment between anticholinergic effect (burden/use) with xerostomia and/or xerophthalmia |
| Thomson WM, Ferguson CA, Janssens BE, Kerse NM, Ting GS, Smith MB. ﻿Xerostomia and polypharmacy among dependent older New Zealanders: a national survey. Age and Ageing. 2021;50:248-251 | No association assessment between anticholinergic effect (burden/use) with xerostomia and/or xerophthalmia |
| Campbell NL, Hines, L, Epstein AJ, Walker D, Lockefeer A, Shiozawa A. A 12-Year Retrospective Study of the Prevalence of Anticholinergic Polypharmacy and Associated Outcomes Among Medicare Patients with Overactive Bladder in the USA. Drugs & aging. 2021; 38(12): 1075-1085 | Population under 65 years included in the study |
