# Supplementary material for: Association between anticholinergic activity and xerostomia and/ or xerophthalmia in the elderly: systematic review
Source: BMC Pharmacol Toxicol. 2022 Dec 21;23:94. doi: 10.1186/s40360-022-00637-8 (PMC9769019; doi:10.1186/s40360-022-00637-8)
Supplement: Supplementary file 2 — Additional file 2: Appendix 2. Quality assessment of included studies (JBI Critical Appraisal tool for Cross-sectional studies, Newcastle-Ottawa Quality Assessment Scale for cohort and case-control studies, Rob 2.0 tool for randomized trials). [file 40360_2022_637_MOESM2_ESM.docx]

**Association between anticholinergic activity with xerostomia and/or xerophthalmia in elderly: a systematic review**

**Authors:**

Prado-Mel E^1^, Ciudad-Gutiérrez P^1^, Rodríguez-Ramallo H^1^ ,Sánchez-Fidalgo S^2^, Santos-Ramos B^1^, Villalba-Moreno AM^1^

1. Hospital Universitario Vírgen del Rocío, (Pharmacy department), Seville, (Andalucía), Spain.

2. University of Seville, (Department of Preventive Medicine and Public Health), Seville, (Andalucía), Spain.

**Corresponding author**: Sánchez-Fidalgo S, Avenida Dr Fedriani SN, Sevilla, CP: 41009, telephone: 954551771; [fidalgo@us.es](mailto:fidalgo@us.es)

ORCID 0000-0002-3630-7122

**Appendix 2. Quality assessment of included studies**

**Quality assessment of Cross-sectional studies by JBI Critical Appraisal**

|  | Thomson WM. 1993 | Katz IR. 1988 | Desoutter A. 2012 | Kersten H. 2012 | Tiisanoja A. 2017 | Inkeri NM. 2019 |
| --- | --- | --- | --- | --- | --- | --- |
| 1. Were the criteria for inclusion in the sample clearly defined? | Y | Y | Y | Y | Y | U |
| 2. Were the study subjects and the setting described in detail? | Y | Y | Y | Y | Y | Y |
| 3. Was the exposure measured in a valid and reliable way? | U | U | U | Y | Y | U |
| 4. Were objective, standard criteria used for measurement of the condition? | U | U | U | Y | Y | U |
| 5. Were confounding factors identified? | N | N | N | Y | Y | N |
| 6. Were strategies to deal with confounding factors stated? | N | N | N | N | Y | N |
| 7. Were the outcomes measured in a valid and reliable way? | U | U | U | Y | Y | U |
| 8. Was appropriate statistical analysis used? | U | U | Y | Y | Y | U |
| Overall appraisal: (include/exclude/Seek further info) | include | include | include | include | include | include |
| Y=Yes; N=No; U=Unclear; NA=Not Applicable; |  |  |  |  |  |  |
|  |  |  |  |  |  |  |

**Quality assessment of Cohort Studies by Newcastle-Ottawa Quality Assessment Scale.**

|  |  | |  | | |  | |  |  | | | | |  | |  |
| --- | --- | --- | --- | --- | --- | --- | --- | --- | --- | --- | --- | --- | --- | --- | --- | --- |
|  | |  | |  |  | |  | | | **Ness J. 2006** | | | **Rudolph JL. 2008** | |  |  |
| SELECTION(4*) | | 1) Representativeness of the exposed cohort | | | a) truly representative of the average _______________ (describe) in the community * | | | | |  | | |  | |  |  |
|  |  |  |  |  | b) somewhat representative of the average ______________ in the community * | | | | | "Patients with anticholinergics drugs" X(*) | | | "Those with ARS score or 1 or 2, and those with an ARS score of 3 of higher" X(*) | |  |  |
|  |  |  |  |  | c) selected group of users eg nurses, volunteers | | | | |  | | |  | |  |  |
|  |  |  |  |  | d) no description of the derivation of the cohort | | | | |  | | |  | |  |  |
|  |  | 2) Selection of the non exposed cohort | | | a) drawn from the same community as the exposed cohort * | | | | | X(*) | | | X(*) | |  |  |
|  |  |  |  |  | b) drawn from a different source | | | | |  | | |  | |  |  |
|  |  |  |  |  | c) no description of the derivation of the non exposed cohort | | | | |  | | |  | |  |  |
|  |  | 3) Ascertainment of exposure | | | a) secure record (eg surgical records) * | | | | | X(*) | | | X(*) | |  |  |
|  |  |  |  |  | b) structured interview * | | | | |  | | |  | |  |  |
|  |  |  |  |  | c) written self report | | | | |  | | |  | |  |  |
|  |  |  |  |  | d) no description | | | | |  | | |  | |  |  |
|  |  | 4) Demonstration that outcome of interest was not present at start of study | | | a) yes * | | | | |  | | |  | |  |  |
|  |  |  |  |  |  |  |  |  |  |  |  |  |  |  |  | |
|  |  |  |  |  | b) no | | | | | X | | | X | |  | |
|  |  |  |  |  |  |  |  |  |  |  |  |  |  |  |  | |
| COMPARABILITY (2*) | | 1) Comparability of cohorts on the basis of the design or analysis | | | a) study controls for _____________ (select the most important factor) * | | | | | "Patients without anticholinergics drug" X(*) | | | "Those with an ARS score of 0" X(*) | |  | |
|  |  |  |  |  |  |  |  |  |  |  |  |  |  |  |  | |
|  |  |  |  |  | b) study controls for any additional factor * (This criteria could be modified to indicate specific control for a second important factor.) | | | | |  | | |  | |  | |
|  |  |  |  |  |  |  |  |  |  |  |  |  |  |  |  | |
| OUTCOME (3*) | | 1) Assessment of outcome | | | a) independent blind assessment * | | | | |  | | |  | |  | |
|  |  |  |  |  | b) record linkage * | | | | | X(*) | | | X(*) | |  | |
|  |  |  |  |  | c) self report | | | | |  | | | X | |  | |
|  |  |  |  |  | d) no description | | | | |  | | |  | |  | |
|  |  | 2) Was follow-up long enough for outcomes to occur | | | a) yes (select an adequate follow up period for outcome of interest) * | | | | | X(*) | | |  | |  | |
|  |  |  |  |  | b) no | | | | |  | | | X | |  | |
|  |  | 3) Adequacy of follow up of cohorts | | | a) complete follow up - all subjects accounted for * | | | | |  | | |  | |  | |
|  |  |  |  |  | b) subjects lost to follow up unlikely to introduce bias - small number lost - > ____ % (select an adequate %) follow up, or description provided of those lost) * | | | | |  | | |  | |  | |
|  |  |  |  |  | c) follow up rate < ____% (select an adequate %) and no description of those lost | | | | |  | | |  | |  | |
|  |  |  |  |  | d) no statement | | | | | X | | | X | |  | |
| **Total Score** | | | | | | | | | | ****** (6) | | | ***** (5) | |  | |
|  | |  | |  |  | |  | | |  |  |  |  | |  | |

**Quality assessment of Case-Control Study by New Castle Ottawa Quality Assesment Scale.**

|  |  |  |  |  |  |  |
| --- | --- | --- | --- | --- | --- | --- |
|  |  |  |  |  |  | **Lavrador M. 2021** |
| SELECTION (4*) | 1)Is the case definition adequate? | | a) yes, with independent validation * | | |  |
|  |  |  | b) yes, eg record linkage or based on self reports | | |  |
|  |  |  | c) no description | | | X |
|  | 2) Representativeness of the cases | | a) consecutive or obviously representative series of cases * | | | X(*) |
|  |  |  | b) potential for selection biases or not stated | | |  |
|  | 3) Selection of Controls | | a) community controls * | | |  |
|  |  |  | b) hospital controls | | | X |
|  |  |  | c) no description | | |  |
|  | 4) Definition of Controls | | a) no history of disease (endpoint) * | | | X(*) |
|  |  |  | b) no description of source | | |  |
| COMPARABILITY (2*) | 1) Comparability of cases and controls on the basis of the design or analysis | | a) study controls for _______________ (Select the most important factor.) * | | | X(*) "Anticholinergic burden" |
|  |  |  |  |  |  |  |
|  |  |  |  |  |  |  |
|  |  |  | b) study controls for any additional factor * (This criteria could be modified to indicate specific control for a second important factor.) | | |  |
|  |  |  |  |  |  |  |
|  |  |  |  |  |  |  |
| EXPOSURE (3*) | 1) Ascertainment of exposure | | a) secure record (eg surgical records) * | | | X(*) |
|  |  |  | b) structured interview where blind to case/control status * | | |  |
|  |  |  | c) interview not blinded to case/control status | | |  |
|  |  |  | d) written self report or medical record only | | |  |
|  |  |  | e) no description | | |  |
|  | 2) Same method of ascertainment for cases and controls | | a) yes * | | | X(*) |
|  |  |  | b) no | | |  |
|  | 3) Non-Response rate | | a) same rate for both groups * | | |  |
|  |  |  | b) non respondents described | | | X |
|  |  |  | c) rate different and no designation | | |  |
| **Total Score** | | | | | | ***** (5) |

**Rob 2.0. A revised tool to assess risk of bias in randomized trials.**

|  | **Kertsten H. 2013** | | |  |
| --- | --- | --- | --- | --- |
| Bias domain and signalling question* | **Response options** | | |  |
|  | Lower risk of bias | Higher risk of bias | Other |  |
| **Bias arising from the randomisation process** | | | |  |
| 1.1 Was the allocation sequence random? | Y |  |  |  |
| 1.2 Was the allocation sequence concealed until participants were errolled and assigned to interventions? | Y |  |  |  |
| 1.3 Did baseline differences between interventions groups suggest a problem with the randomisation process? | N |  |  |  |
| Risk of bias judgment (low/high/some concerns) **LOW RISK** | | | |  |
| **Bias due to deviations from intended interventions** | | | |  |
| 2.1 Were participants aware of their assigned intervention during the trial? |  | PY |  |  |
| 2.2 Were carers and people delivering the interventions aware of participants´assigned intervention during the trial? |  | Y |  |  |
| 2.3 If Y/PY/NI to 2.1 or 2.2: Were there deviations from the intended intervention that arose because of the trial context? | PN |  |  |  |
| 2.4 If Y/PY/NI to 2.3: Were these desviations likely to have affected the outcome? |  |  | NA |  |
| 2.5 If Y/PY to 2.4: Were these deviations from intended intervention balanced between groups? |  |  | NA |  |
| 2.6 Was an appropiate analysis used to estimate the effect of assignment to intervention? | Y |  |  |  |
| 2.7 If N/PN/NI to 2.6: Was there potential for a sustantial impact (on the result) of the failure to analyse participants in the group to which they were randomised? |  |  | NA |  |
| Risk-of-bias judgment (low/high/some concerns)  **LOW RISK** | | | |  |
| **Bias due to missing outcome data** | | | |  |
| 3.1 Were data for this outcome available for all, or nearly all, participants randomised? | Y |  |  |  |
| 3.2 If N/PN/NI to 3.1: Is there evidence that the result was not biased by missing outcome data? |  |  | NA |  |
| 3.3 If N/PN to 3.2: Could missingness in the outcome depend on its true value? |  |  | NA |  |
| 3.4 If Y/PY/NI to 3.3: It is likely that missingness in the outcome depended on its true value? |  |  | NA |  |
| Risk-of-bias judgment (low/high/some concerns) **LOW RISK** | | | |  |
| **Bias in measurement of the outcome** | | | |  |
| 4.1 Was the method of measuring the outcome inappropiate? | N |  |  |  |
| 4.2 Could measurement or ascertainment of the outcome have differed between intervention groups? | PN |  |  |  |
| 4.3 If N/PN/NI to 4.1 and 4.2: Were outcome assesors aware of the intervention received by study participants? |  |  | NI |  |
| 4.4 If Y/PY/NI to 4.3: Could assessment of the outcome have been influenced by knowledge of intervention received? | PN |  |  |  |
| 4.5 If Y/PY/NI to 4.4: Is it likely that assessment of the outcome was influenced by knowledge of intervention received? |  |  | NA |  |
| Risk-of-bias judgment (low/high/some concerns)  **LOW RISK** | | | |  |
| 5.1 Were the data that produced this result analysed in accordance with a prespecified analysis plan that was finalised before unblinded outcome data were available for analysis? | PY |  |  |  |
| Is the numerical result being assessed likely to have been selected, on the basis of the results, from |  |  |  |  |
| 5.2 …multiple eligible outcome measurements (eg, scales, definitions, time points) within the outcome domain? | PN |  |  |  |
| 5.3 ….multiple elegible analyses of the data? | PN |  |  |  |
| Risk-of-bias judgment (low/high/some concerns)  **LOW RISK** | | | |  |
| **Overall bias** | | | |  |
| Risk-of-bias judgment (low/high/some concerns)  **LOW RISK** | | | |  |
| Y=yes; PY=probably yes; PN=probably no; N=no; NA= not applicable; NI: no information. *Signalling questions for bias due to deviations from intended interventions relate to the effect of assignment to intervention | | | |  |
|  |  |  |  |  |

**References:**

1. Desoutter A, Soudain-Pineau M, Munsch F, Mauprivez C, Dufour T, Coeuriot JL. Xerostomia and medication: a cross-sectional study in long-term geriatric wards. J Nutr Health Aging. 2012;16(6):575-9.
2. Inkeri NM, Karjalainen M, Haanpää M, Kautiainen H, Saltevo J, Mäntyselkä P, Tiihonen M. Anticholinergic drug use and its association with self-reported symptoms among older persons with and without diabetes. J Clin Pharm Ther. 2019;44(2):229-235.
3. Katz IR, Stoff D, Muhly C, Bari M. Identifying persistent adverse effects of anticholinergic drugs in the elderly. J Geriatr Psychiatry Neurol. 1988;1(4):212-7.
4. Kersten H, Molden E, Tolo IK, Skovlund E, Engedal K, Wyller TB. Cognitive effects of reducing anticholinergic drug burden in a frail elderly population: a randomized controlled trial. J Gerontol A Biol Sci Med Sci. 2013;68(3):271-8.
5. Kersten H, Molden E, Willumsen T, Engedal K, Bruun Wyller T. Higher anticholinergic drug scale (ADS) scores are associated with peripheral but not cognitive markers of cholinergic blockade. Cross sectional data from 21 Norwegian nursing homes. Br J Clin Pharmacol. 2013;75(3):842-9.
6. Lavrador M, Cabral AC, Figueiredo IV, Veríssimo MT, Castel-Branco MM, Fernandez-Llimos F. Size of the associations between anticholinergic burden tool scores and adverse outcomes in older patients. Int J Clin Pharm. 2021. 43(1):128-136.
7. Ness J, Hoth A, Barnett MJ, Shorr RI, Kaboli PJ. Anticholinergic medications in community-dwelling older veterans: prevalence of anticholinergic symptoms, symptom burden, and adverse drug events. Am J Geriatr Pharmacother. 2006;4(1):42-51.
8. Rudolph JL, Salow MJ, Angelini MC, McGlinchey RE. The anticholinergic risk scale and anticholinergic adverse effects in older persons. Arch Intern Med. 2008;168(5):508-13.
9. Thomson WM, Brown RH, Williams SM. Medication and perception of dry mouth in a population of institutionalised elderly people. N Z Med J. 1993;106(957):219-21.
10. Tiisanoja A, Syrjälä AM, Komulainen K, Lampela P, Hartikainen S, Taipale H, Knuuttila M, Ylöstalo P. Anticholinergic burden and dry mouth among Finnish, community-dwelling older adults. Gerodontology. 2018;35(1):3-10.
