# Supplementary material for: Association between anticholinergic activity and xerostomia and/ or xerophthalmia in the elderly: systematic review
Source: BMC Pharmacol Toxicol. 2022 Dec 21;23:94. doi: 10.1186/s40360-022-00637-8 (PMC9769019; doi:10.1186/s40360-022-00637-8)
Supplement: Supplementary file 3 — Additional file 3: Appendix 3. Tools used for measure Xerostomia and Xerophthalmia (Description of the methods used in the studies to measure xerostomia and/or xerophthalmia). [file 40360_2022_637_MOESM3_ESM.docx]

**Association between anticholinergic activity with xerostomia and/or xerophthalmia in elderly: a systematic review**

**Authors:**

Prado-Mel E^1^, Ciudad-Gutiérrez P^1^, Rodríguez-Ramallo H^1^ ,Sánchez-Fidalgo S^2^, Santos-Ramos B^1^, Villalba-Moreno AM^1^

1. Hospital Universitario Vírgen del Rocío, (Pharmacy department), Seville, (Andalucía), Spain.

2. University of Seville, (Department of Preventive Medicine and Public Health), Seville, (Andalucía), Spain.

**Corresponding author**: Sánchez-Fidalgo S, Avenida Dr Fedriani SN, Sevilla, CP: 41009, telephone: 954551771; [fidalgo@us.es](mailto:fidalgo@us.es)

ORCID 0000-0002-3630-7122

| **Study.year** | **Xerostomia methods** | **Xerophthalmia methods** |
| --- | --- | --- |
| Thomson WM. 1993 | - Question: How often do you feel dry?  - Answers: Always or frequently vs occasionally or never | No tested |
| Katz IR. 1988 | - Question: Is presence or absence of dry mouth?  - Answers: present or absence | No tested |
| Ness J. 2006 | - Question: In the past 4 weeks, how much have you been bothered by dry mouth  - Answer: scale symptom burden (0= not at all bothersome to 4 =very much bothersome). An anticholinergic was considered absent if the rating was 0. | - Question: In the past 4 weeks, how much have you been bothered by dry eyes?  - Answer: scale symptom burden (0= not at all bothersome to 4 =very much bothersome). An anticholinergic was considered absent if the rating was 0.  -Eye tears prescription registration |
| Rudolph JL. 2008 | - Retrospective cohort: review of systems in the medical record. Anticholinergic adverse effects identified (falls, dry mouth, dry eyes, dizziness, confusion and constipation) | - Retrospective cohort: review of systems in the medical record. Anticholinergic adverse effects identified (falls, dry mouth, dry eyes, dizziness, confusion and constipation) |
|  | - Prospective cohort: modified review of systems (20 questions) with anticholinergic adverse effects identified (falls, dry mouth, dry eyes, dizziness, confusion and constipation). | - Prospective cohort: modified review of systems (20 questions) with anticholinergic adverse effects identified (falls, dry mouth, dry eyes, dizziness, confusion and constipation). |
| Desoutter A. 2012 | - Question 1: Do you suffer dry mouth (Yes/No)  - Question 2: Do you often thirsty (Yes/No)  Xerostomia is present if the answer for the two question are yes | No tested |
| Kersten H. 2012 | - Salivary secretion: swab technique | No tested |
| Kersten H. 2013 | - Salivary secretion: swab technique | No tested |
| Tiisanoja A. 2017 | - Salivary secretion unstimulated and stimulated: draining method  - Question: How often does you mouth feel dry?  - Answer: never/occasionally vs often | No tested |
| Inkeri NM. 2019 | - Participant were asked to assess whether they had experienced dry mouth.  - The participants ranked the severity of each symptom with a numerical rating scale from 0 to 10. | - Participant were asked to assess whether they had experienced dry eye.  - The participants ranked the severity of each symptom with a numerical rating scale from 0 to 10. |
| Lavrador M. 2021 | - Salivary secretion: swab technique | - Tear secretion: Schirmer test |

**Appendix 3. Tools used for measure Xerostomia and Xerophthalmia**
