# Supplementary material for: Association between anticholinergic activity and xerostomia and/ or xerophthalmia in the elderly: systematic review
Source: BMC Pharmacol Toxicol. 2022 Dec 21;23:94. doi: 10.1186/s40360-022-00637-8 (PMC9769019; doi:10.1186/s40360-022-00637-8)
Supplement: Supplementary file 4 — Additional file 4: Supplementary table S1. Alternative causes of Xerophthalmia (Confounding factors for Xerophthalmia considered in the studies). [file 40360_2022_637_MOESM4_ESM.docx]

**Association between anticholinergic activity with xerostomia and/or xerophthalmia in elderly: a systematic review**

**Authors:**

Prado-Mel E^1^, Ciudad-Gutiérrez P^1^, Rodríguez-Ramallo H^1^, Sánchez-Fidalgo S^2^, Santos-Ramos B^1^, Villalba-Moreno AM^1^

1. Hospital Universitario Vírgen del Rocío, (Pharmacy department), Seville, (Andalucía), Spain.

2. University of Seville, (Department of Preventive Medicine and Public Health), Seville, (Andalucía), Spain.

**Corresponding author**: Sánchez-Fidalgo S, Avenida Dr Fedriani SN, Sevilla, CP: 41009, telephone: 0034 954551771; [fidalgo@us.es](mailto:fidalgo@us.es)

ORCID 0000-0002-3630-7122

**Supplementary Table S1. Alternative causes of Xerophthalmia**

| **Study. year** | **Diabetes** | **Sjogrën Disease** | **Rheumatoid arthritis** | **Polypharmacy^a^** | **Ophthalmic Surgery** |
| --- | --- | --- | --- | --- | --- |
| Ness J. 2006 | - | - | - | Inclusion criteria: active prescriptions for ≥5 drugs.  Mean number of drugs: 9,2 ± 3,9  Mean number of total prescriptions was significantly higher in the group using at least 1 anticholinergic drugs vs the group not using anticholinergic drugs (10.3 vs 8.8, (p<0,001)^b^ | - |
| Rudolph JL. 2008 | - | - | - | Mean number of drugs:  - Retrospective cohort: 7,9 ± 2,8  - Prospective cohort: 9 ± 4,5  The total number of medications prescribed was used as surrogated form medical comorbidity | - |
| Inkeri NM. 2019 | (CCI^c^ , 0.4-0.5 (0.7) No diabetes vs (1.7-1.9 (1.1)) diabetes patients, (p<0,001))^d^. The use of anticholinergic drug was associated with an increased risk of xerostomia in the no diabetes group  The use of anticholinergic drugs was associated with an increased risk of xerophthalmia in the diabetes group. | - | Musculoskeletal disorder between ARS>0 vs ARS=0 (65% vs 46,86%,(p<0,001))^d^  Musculoskeletal disorder between Diabetes group vs no diabetes group (54,65% vs 44%,(p=0,081)^d^ | Polypharmacy was more prevalent in patients with anticholinergic burden in both groups: No diabetes: 2,0 ± 2,6 vs 6,0 ± 3,3; Diabetes: 3,6 ± 3,1 vs 7,0 ± 4,4 | - |
| Lavrador M. 2021 | No data. Multivariable analyses for the score of each anticholinergic burden scale were performed used diabetes, among others, as covariates of the analysis. | - | No data. Multivariable analyses for the score of each anticholinergic burden scale were performed used autoimmune disease, among others, as covariates of the analysis | - | - |

^a^Polypharmacy was defined as >5 drugs; ^b^ Chi square test; ^c^ CCI: Charlson Comorbidity Index (Mean (Standard deviation); ^d^ANOVA test; ^e^ARS: Anticholinergic Risk Scale;
