# Supplementary material for: Association between anticholinergic activity and xerostomia and/ or xerophthalmia in the elderly: systematic review
Source: BMC Pharmacol Toxicol. 2022 Dec 21;23:94. doi: 10.1186/s40360-022-00637-8 (PMC9769019; doi:10.1186/s40360-022-00637-8)
Supplement: Supplementary file 5 — Additional file 5: Supplementary table S2. Alternative causes of Xerostomia (Confounding factors for Xerostomia considered in the studies). [file 40360_2022_637_MOESM5_ESM.docx]

**Association between anticholinergic activity with xerostomia and/or xeropthalmia in elderly: a systematic review**

**Authors:**

Prado-Mel E^1^, Ciudad-Gutiérrez P^1^, Rodríguez-Ramallo H^1^, Sánchez-Fidalgo S^2^, Santos-Ramos B^1^, Villalba-Moreno AM^1^

1. Hospital Universitario Vírgen del Rocío, (Pharmacy department), Seville, (Andalucía), Spain.

2. University of Seville, (Department of Preventive Medicine and Public Health), Seville, (Andalucía), Spain.

**Corresponding author**: Sánchez-Fidalgo S, Avenida Dr Fedriani SN, Sevilla, CP: 41009, telephone: 0034 954551771; [fidalgo@us.es](mailto:fidalgo@us.es)

ORCID 0000-0002-3630-7122

**Supplementary Table S2. Alternative causes of xerostomia**

| **Study. year** | **Diabetes** | **Sjogrën Disease** | **Rheumatoid arthritis** | **Polypharmacy^a^** | **Radiotherapy** |
| --- | --- | --- | --- | --- | --- |
| Thomson WM. 1993 | - | - | - | Mean number drug: 3,6 ± 2,37 | - |
| Katz IR. 1988 | No data. Patients for whom anticholinergic-related symptoms could be attributed to known somatic disease were excluded from data analysis | No data. Patients for whom anticholinergic-related symptoms could be attributed to known somatic disease were excluded from data analysis | No data. Patients for whom anticholinergic-related symptoms could be attributed to known somatic disease were excluded from data analysis | Mean number of drugs: 5,8 ± 2,9. Range: 1-14. |  |
| Ness J. 2006 | - | - | - | Inclusion criteria: active prescriptions for ≥5 drugs.  Mean number of drugs: 9,2 ± 3,9  Mean number of total prescriptions was significantly higher in the group using at least 1 anticholinergic drug vs not using (10.3 vs 8.8(p<0,001))^b^ | - |
| Rudolph JL. 2008 | - | - | - | Mean number of drugs:  - Retrospective cohort: 7,9 ± 2,8  - Prospective cohort: 9 ± 4,5  The total number of medications prescribed was used as surrogated form medical comorbidity | - |
| Desoutter A. 2012 | - | - | - | OR(95% IC): 0,97 ( 0,93-1)^c^.  Total number of medications was not significantly associated with xerostomia | - |
| Kersten H. 2012 | No data. Co-morbidity was assessed by the CCI^d^ | - | No data. Co-morbidity was assessed by the CCI^d^ | Scheduled drug, median (IQR^e^):  (ADS=3): 8 (6-10)  (ADS=4): 9 (7-10)  (ADS=5): 10.5 (8-13)  (ADS=6):12 (9.5-5.5) | - |
| Kersten H. 2013 | No data. Co-morbidity was assessed by the CCI^d^ | - | No data. Co-morbidity was assessed by the CCI^d^ | Scheduled drug, Median (IQR^e^)  Intervention group:10 (7-13)  Control group: 9 (6-11) | - |
| Tiisanoja A. 2017 | RR=2.7 (IC 95%: 1.4-5.1)^f^  Diabetes was included as individual variable.  All the models were adjusted diabetes among others, and the FCI^g^ | - | RR(95% IC)= 0,8 (0,3-2,4)^f^  Reumatoid arthritis was included as individual variable.  All the models were adjusted for rheumatoid disease, among others, and the FCI^g^ | Mean number of drugs: (ADS=0): 4.2 ± 2.6  (ADS=1-2): 7.2 ± 3.4  (ADS≥3):10.2 ± 4.3  An additional adjustment was made for the total number of medications, among others, for all outcomes. | - |
| Inkeri NM. 2019 | (CCI^d^ , 0.4-0.5 (0.7) No diabetes vs (1.7-1.9 (1.1)) diabetes patients, (p<0,001))^h^. The use of anticholinergic drug was associated with an increased risk of xerostomia in the no diabetes group. | - | Musculoskeletal disorder between ARS>0 vs ARS=0 (65% vs 46,86%,(p<0,001))^h^  Musculoskeletal disorder between Diabetes group vs no diabetes group (54,65% vs 44%,(p=0,081)^h^ | Polypharmacy was more prevalent in patients with anticholinergic burden in both groups: No diabetes: 2,0 ± 2,6 vs 6,0 ± 3,3; Diabetes: 3,6 ± 3,1 vs 7,0 ± 4,4 | - |
| Lavrador M. 2021 | No data. Multivariable analyses for the score of each anticholinergic burden tool were performed used diabetes, among others, as covariates of the analysis | - | No data. Multivariable analyses for the score of each anticholinergic burden tool were performed used autoimmune disease, among others, as covariates of the analysis | - | - |

^a^ Polypharmacy was defined as >5 drugs; ^b^ Chi square test; ^c^ OR=Odds Ratio, multivariate logistic regression; ^d^CCI: Charlson Co-morbidity Index; ^e^IQR : Interquartile range; ^f^RR=Relative risk, poisson regression; ^g^ FCI: Functional Co-morbidity Index; ^h^ANOVA test.
